# Fibroblast growth factor-21 and Visfatin as potential predictors for metabolic risk factors in obese children

**DOI:** 10.1038/s41598-024-51394-z

**Published:** 2024-01-12

**Authors:** Sahar A. El-Masry, Maisa Nasr Farid, Nayera E. Hassan, Muhammad Al-Tohamy Soliman, Lamis H Mekkawy, Galal Ismail Elashry, Safa N. Abd El-Fattah

**Affiliations:** 1 Biological Anthropology Department, Medical Research and Clinical Studies Institute, National Research Centre, 33 El-Buhouth St., Dokki, Giza, 12622 Egypt; 2 Faculty of PostGraduate Childhood Studies, Ain Shams University, Cairo, Egypt; 3 Clinical and Chemical Pathology Department, Medical Research and Clinical Studies Institute, National Research Centre, Giza, Egypt

**Keywords:** Health care, Medical research, Risk factors

## Abstract

Fibroblast growth factor-21 (FGF-21) and Visfatin are associated with obesity. However; reviewing the literature; no studies were found to assess their role as potential markers for the metabolic disorders related to obesity in children. Assess the relations between serum FGF-21 and Visfatin with obesity and its metabolic disorders, and their use as potential predictors for metabolic risk factors in a sample of Egyptian obese children. This cross-sectional study included 111 Egyptian children (45 males and 66 females); aged 6–10 years to avoid the effect of puberty (prepubertal). The exclusion criteria (by full History taking and clinical examination) were the presence of any sign of puberty according to Tanner stage, the presence of identified causes of obesity (genetic syndromes, chromosomal or endocrinal disorders), chronic diseases (cardiovascular, gastrointestinal, and respiratory), or drug use like steroids; that would interfere with the type of obesity and affect the normal growth of the children. Also, any child with a BMI between 85 and 95th percentiles (overweight) was excluded from the study. All participating obese children were suffering from exogenous simple obesity. They were classified according to their body mass index (BMI) percentiles into 72 obese (BMI ≥ 95th), and 39 control non-obese ones (BMI > 15th to < 85th), based on the Egyptian Growth Charts for children and adolescents. Ethical approvals were granted from both the Ethics Committee of the “National Research Centre” and the “Faculty of Postgraduate Childhood Studies” (Approval No. 17/125). Also, informed written consent was taken from either of the parents and assent from the participating children. They were subjected to blood pressure assessment, anthropometric measurements (weight [Wt], height [Ht], BMI, waist [WC], and hip [HC] circumferences), and laboratory evaluation (Visfatin, FGF-21, LDL, HDL, TG, cholesterol, fasting glucose, insulin, and calculation of HOMA-IR). Mann–Whitney test and Spearman’s correlation test were applied. Obese children had significantly higher values than control ones regarding all the studied clinical (SBP, DBP), anthropometric parameters (Wt, Ht, BMI, WC, and HC), FBG, Insulin, HOMA-IR, Visfatin, and FGF-21, and had significantly lower values regarding HDL and Cholesterol. Among obese children, both FGF-21 and Visfatin had significant negative correlations with BMI and HC. At the same time, serum FGF-21 had a highly significant positive correlation with HDL. Visfatin and FGF-21 had highly significant positive correlations with each other. In the control group, both serum Visfatin or FGF-21 had insignificant correlations with each other and with all the studied clinical and anthropometric parameters. FGF-21 and Visfatin are related to the obesity markers, but they cannot be used as potential predictors for metabolic disturbance in obese prepubertal children; both had insignificant correlations with the metabolic risk factors.

## Introduction

Obesity is a major risk factor for several chronic diseases. It is associated with many systemic micro inflammation, as the major risk factor for different metabolic syndromes, as dyslipidaemia and type 2- diabetes, due to the secretion of specific pro-inflammatory peptides from the visceral adipose tissue^1^. In Egypt, Hassan et al.^2^, ported that the prevalence of obesity among Egyptian school students was 8.0% in 2011; and increase to 19.5% in 2018. The rapid increase in the prevalence of obesity among children and adolescents, made it the most important worldwide problem of the twenty-first century. So, it became necessary to encounter new biomarkers for both obesity and its associated metabolic disorders^3^.

Adipokines secretions, of the adipose tissue, are regarded as mechanisms relating obesity to comorbidities. However, such adipokines regulate a number of systemic processes, as inflammations, nutrient metabolism, food intake and insulin sensitivity^4^. Of the adipokines that may be responsible for such co- morbidities is the adipokines Visfatin through a variety of mechanisms^5^. It has been suggested that Visfatin might have both endocrine and paracrine effects^6^, mostly related to obesity and insulin sensitivity although there are important discrepancies in the literature^7^.

Fibroblast growth factor-21 (FGF-21); a stress-inducible hormone primarily produced by the liver in response to ketosis, and crosses the blood–brain barrier (as metabolic stressful stimuli). Such FGF-21 activates numerous brain areas including “PVN” the hypothalamic paraventricular nucleus, in turn it plays a role in regulating of the hypothalamic pituitary adrenal (HPA) axis^8^. Moreover, FGF-21 was initially proposed as a lipolysis inducer in fat tissue. Animal studies have shown that FGF-21, when given to diabetic transgenic mice, and hence its level is overexpressed in their bodies, and as a result it lowers their blood glucose and triacylglycerol levels, thus it protects them from diet-induced obesity^9^. Higher levels of serum FGF-21 were found to be related to obesity in children^10^, and with disturbed metabolic parameter such as dyslipidaemia and insulin resistance^11^. Moreover, FGF-21 has been proposed as a possible biomarker for the components of the metabolic syndrome (Mets), as well as Type II diabetes mellitus (TII DM)^12^.

Reviewing literature, no studies were found to assess the relation between FGF-21 and Visfatin as potential markers for obesity and its metabolic disorders in both children and adolescents. Therefore, the purpose of this study was to assess the relations between both serum FGF-21 and Visfatin with the obesity and its metabolic disorders, and their use as potential predictors for metabolic risk factors in a sample of Egyptian obese and non-obese children.

## Subjects and methods

### Subjects

The present study was a cross-sectional one that was conducted in the “Visceral Obesity and Growth Disturbances Management clinic” in “Medical Research Centre of Excellence (MRCE)”, National Research Centre (Egypt), during the period between December 2018 and February 2021. It was conducted on 2 groups of children N = 111; with their ages ranging 6–10 years (45 males; mean age 8.74 ± 1.60 years and 66 females; with mean age 8.78 ± 1.73 years to exclude the possible effects of puberty. The exclusion criteria (by full History taking and clinical examination) were the presence of any sign of puberty according to Tanner stage, presence of identified causes of obesity (genetic syndromes, chromosomal or endocrinal disorders), chronic diseases (cardiovascular, gastrointestinal, and respiratory), or drug use like steroids; that would interfere with the type of obesity and affect the normal growth of the children. Also, any child with a BMI between 85 to 95th percentiles (overweight) was excluded from the study. All participating obese children were suffering from exogenous simple obesity. They were classified according to their BMI percentiles into: 72 obese (BMI ≥ 95th), and 39 control non-obese ones (BMI > 15th to < 85th), based on the Egyptian Growth Charts for children and adolescents^13^.

Ethical approval were granted from both the Ethics Committee of the “National Research Centre” (Approval No. 17/125). And that from the Ethics Committee of “Faculty of Postgraduate Childhood Studies”.

Also, after clarifying the main objectives of the research and its conceivable benefits in identifying the risks of obesity on family health, an informed written consent was taken from either of the parents and an assent from the participated children (both signed in and dated).

### Methods

Each child was subjected to blood pressure assessment, anthropometric measurements and laboratory investigations.

- Blood pressure assessment

Using a standardized mercury sphygmomanometer, while sitting in a proper position, both systolic and diastolic blood pressures were measured. Applying appropriate blood pressure cuff that did not encroach on the antecubital space. Three successive readings were measured, and the mean was recorded if the error was satisfactory.

- Anthropometric evaluation

The following anthropometric parameters were recorded, using identical equipment’s and following the recommendations of the “IBP” International Biological Program, including: bodyweight “Wt”, height “Ht”, waist and hip circumferences “WC” and “HC”;^14^.

Digital standing SECA scale balance (Model 707) was used to measure body weight that was recorded to the nearest 0.01 kg. Height, that was recorded to the nearest 0.1 cm, was measured using a wall mounted Holtain Stadiometer. Waist circumference (WC) was done using a non-stretchable plastic measuring tape, all around the body in horizontal position, and at a level midway between the lower rib margin and iliac crest and at the end of normal expiration. The observer held the measuring tape firmly, to ensure a horizontal position on the subject’s body. The WC measure was approximated to the nearest 0.1 cm. Hip circumference (HC) was measured using flexible non-stretchable plastic tape, which was held horizontally around the maximum extension of the buttocks, the reading was approximated to the nearest 0.1 cm. BMI was calculated using the formula: BMI = Weight (kg)/[Height (m^2^)]. According to their BMI percentile; based on the Egyptian Growth Charts for children and adolescents^13^; obesity was diagnosed more than or equal to 95% and healthy weight 15%–less than 85%.

- Laboratory investigations

After fasting for 12 h, a 5 ml venous blood sample (between 9–11 am) was obtained from every child by professional venepuncture staff. After being clotted, it was centrifuged and its serum was obtained to be kept at − 80 °C, to be further assessed. Fasting blood glucose (FBG), insulin, lipid profile, Visfatin and FGF21 were then assessed.

FBG was assessed using GOD-POD enzymatic colorimetric method, and serum insulin was assessed using Enzyme Immunoassay, according to the method of Tietz^15^. HOMA-IR was calculated as follows: “HOMA-IR = fasting glucose (mg/dl) × fasting insulin (μIU/ml)/405”.

The Beckman Coulter/Olympus AU480 Random Access Chemistry Analyzer was used to evaluate the levels of serum lipid. Using quantitative enzymatic colorimetric methods were used to assess both serum Triglycerides level “TG” (test kit code no: SU033, SU034, SU035 (CHEMELEX, S.A., Barcelona), and total cholesterol (kit Ref: 101-0440/101-0526 (CHRONOLAB SYSTEMS, Barcelona). HDL was assessed using kit code no: SU014 (CHEMELEX, S.A., Barcelona). Serum triglycerides, total cholesterol and HDL were assessed according to the method of Tietz^15^. While LDL was assessed using kit REF: 99 06 10 (QUIMICA CLINICA APLICADA S.A., Spain) according to Polvinyl Sulphate method of Demacker et al.^16^. The metabolic disturbances criteria ; were defined by Wasilewski et al.^17^, as elevated systolic or diastolic blood pressure, or disturbed any parameter of lipid profile, or elevated fasting blood glucose, or elevated fasting insulin or increased the homeostatic model assessment of insulin resistance HOMA-IR).

Based on the principle of competitive enzyme immunoassay, the Enzyme Linked Immunosorbent Assay (ELISA) kits were used to assess both serum Visfatin and FGF21 levels.

### Statistical analysis

For the present study, the computer program SPSS version 18 (Statistical package for social science) was used to do all the statistical analyses. The Kolmogorov–Smirnov test was used to examine data normality. Most of the studied variables were not normally distributed (for example: BMI, WC Visfatin and FGF-21), therefor non-parametric tests were used to analyse them.

For all anthropometric and laboratory parameters, the descriptive statistics (mean ± SD) were calculated. Mann–Whitney test was carried out, to reveal group differences (between any two groups of parametric (quantitative) data. The association between either Visfatin or FGF-21 with all the examined parameters was done using Spearman’s correlation. In all analyses, the statistical standard probability *P* < 0.01 is regarded as highly significant and *P* < 0.05 is regarded as statistically significant.

### Ethics approval and consent to participate

The study protocol was conformed to the ethical guidelines of the 1975 Declaration of Helsinki and was approved by the Ethics Committee of both Faculty of Postgraduate Childhood Studies, and the “National Research Centre; Egypt” (Approval No. 17/125). After explaining the promising benefits of the study in ascertaining the impact of obesity on health, informed written consents were obtained from either parent.

## Results

The results of this study have revealed that all the studied parameters; including age, clinical, anthropometric and laboratory variables such as FGF-21 and Visfatin showed insignificant sex differences. Subsequently, both sexes were gathered in one group (with no sex differentiation) to do all statistical analyses.

The obese children had highly significant higher values than control ones regarding all the studied clinical (SBP, DBP) and anthropometric parameters (Wt, Ht, BMI, WC and HC) (Table 1). They also had highly significant higher values regarding HOMA-IR and FGF-21, and significant higher values than control ones regarding FBG, Insulin and Visfatin. Moreover, obese children had highly significant lower value regarding HDL, and significant lower value in Cholesterol (Table 2). There were insignificant differences between obese and control in TG and LDL.

**Table 1:** Comparison of obese and control non obese children regarding the clinical and anthropometric parameters.

Parameters	CONTROL (N = 39)	OBESE (N = 72)	*P*
	Mean ± SD	Mean ± SD	
Age (years)	7.97 ± 1.60	9.20 ± 1.55	0.000**
Blood pressure			
SBP (mmHg)	94.87 ± 9.63	106.97 ± 15.42	0.000**
DBP (mmHg)	58.72 ± 3.19	66.11 ± 10.95	0.000**
Anthropometry			
Weight (Kg)	23.59 ± 8.80	58.29 ± 11.22	0.000**
Height (cm)	120.15 ± 16.70	140.65 ± 11.38	0.000**
BMI(kg/m^2^)	15.73 ± 1.71	29.28 ± 2.55	0.000**
WC (cm)	58.13 ± 7.15	90.31 ± 9.74	0.000**
HC (cm)	65.46 ± 8.87	99.60 ± 13.34	0.000**

N.B.: *p* < 0.05 = Significant differences. *SBP* systolic blood pressure, *DBP* diastolic blood pressure, *BMI* body mass index, *WC* waist circumference, *HC* hip circumference.

*p < 0.05 = significant differences; ** p < 0.01 = highly significant differences.

**Table 2:** Comparison of obese and control non obese children regarding the laboratory investigations.

Parameters	CONTROL (N = 39)	OBESE (N = 72)	*P*
	Mean ± SD	Mean ± SD	
FBG (mg/dl)	86.51 ± 15.54	94.57 ± 16.77	**0**.029*
Insulin(uIU/mL)	5.27 ± 5.12	10.23 ± 11.59	**0**.010*
HOMA-IR	1.08 ± 1.06	2.84 ± 5.25	**0**.007*
Lipid profile			
Cholesterol (mg/dl)	175.33 ± 30.64	161.34 ± 29.45	**0**.030*
TG (mg/dl)	85.05 ± 26.74	88.08 ± 28.79	0.387
HDL (mg/dl)	54.85 ± 14.4	46.18 ± 20.57	**0**.000**
LDL (mg/dl)	68.15 ± 8.86	66.14 ± 9.29	0.320
Visfatin (ng/ml)	2. 60 ± 2.91	3.00 ± 2.80	**0**.011*
FGF-21 (pg/ml)	40.46 ± 31.81	53.90 ± 39.66	**0**.000**

N.B.: *p* < 0.05 = Significant differences. *FBG* fasting blood glucose, *HOMA-IR* homeostasis model assessment- insulin resistance, *TG* triglycerides, *HDL* high-density lipoprotein, *LDL* low-density lipoprotein, *FGF-21* fibroblast growth factor 21.

*p < 0.05 = significant differences; ** p < 0.01 = highly significant differences.

Table 3 shows Spearman’s correlation analysis between both Visfatin and FGF-21 and the studied variables among the obese group children. In Serum Visfatin had significant negative correlations with BMI and HC only. While serum FGF-21 had highly significant negative correlation with BMI, significant negative correlation with HC, and highly significant positive correlation with HDL. Visfatin and FGF-21 had highly significant positive correlations with each other. There were insignificant correlations between either Visfatin or FGF-21with any of the following laboratory investigations: FBG, insulin, HOMA, Triglycerides, Cholesterol and LDL.

**Table 3:** Spearman’s correlation of Visfatin and FGF-21 with clinical and anthropometric parameters among obese children.

Parameters	Visfatin ng/ml		FGF-21 pg/ml	
	r	*P* value	r	*P* value
Age (years)	− 0.028	0.818	− 0.067	0.583
SBP (mmHg)	− 0.100	0.405	− 0.026	0.834
DBP (mmHg)	0.021	0.864	− 0.001	0.994
BMI (kg/m2)	− **0**.**288**	**0**.015*	− **0**.**308**	**0**.009**
WC (cm)	− 0.121	0.314	− 0.075	0.538
HC (cm)	− **0**.**283**	**0**.017*	− **0**.**263**	**0**.028*
FBG (mg/dl)	0.019	0.873	0.033	0.786
Insulin (uIU/mL)	0.019	0.878	0.075	0.543
HOMA-IR	0.017	0.891	0.052	0.672
Cholesterol (mg/dl)	− 0.209	0.080	− 0.121	0.317
TG (mg/dl)	0.100	0.407	0.234	0.051
HDL (mg/dl)	0.227	0.057	**0**.**316**	**0**.008**
LDL (mg/dl)	− 0.008	0.945	0.012	0.922
Visfatin (ng/ml)			**0**.**738**	**0**.**000**
FGF-21 (pg/ml)	**0**.**738**	**0**.**000**		

N.B.: *p* < 0.01 = highly significant differences, *p* < 0.05 = Significant differences. *SBP* systolic blood pressure, *DBP* diastolic blood pressure, *NC* neck circumference, *WC* waist circumference, *HC* hip circumference, *FBG* fasting blood glucose, *HOMA-IR* homeostasis model assessment-insulin resistance, *TG* triglycerides, *HDL* high-density lipoprotein, *LDL* low-density lipoprotein, *FGF-21* fibroblast growth factor 21.

*p < 0.05 = significant differences; ** p < 0.01 = highly significant differences.

While Spearman’s correlation analysis among control group (Table 4), revealed that both serum Visfatin or FGF-21 had insignificant positive correlations with each other and with all the studied clinical and anthropometric parameters. Serum FGF-21 had significant positive correlation with Cholesterol, while Serum Visfatin had significant positive correlation with Cholesterol and HDL, and significant negative correlation with FBG.

**Table 4:** Spearman’s correlation of Visfatin and FGF-21 with clinical and anthropometric parameters among *control* children.

Parameters	Visfatin ng/ml		FGF-21 pg/ml	
	r	*P* value	r	*P* value
Age (years)	− 0.176	0.283	− 0.143	0.385
SBP (mmHg)	0.032	0.849	− 0.179	0.276
DBP (mmHg)	0.097	0.556	− 0.185	0.260
BMI (kg/m2)	0.079	0.632	0.099	0.550
WC (cm)	− 0.102	0.535	0.046	0.779
HC (cm)	− 0.129	0.434	− 0.007	0.968
FBG (mg/dl)	− **0**.**357**	**0**.026*	0.005	0.976
Insulin (uIU/mL)	− 0.075	0.648	0.188	0.252
HOMA-IR	− 0.088	0.593	0.222	0.174
Cholesterol (mg/dl)	**0**.**375**	**0**.019*	**0**.**327**	**0**.042*
TG (mg/dl)	− 0.103	0.531	0.035	0.834
HDL (mg/dl)	**0**.**383**	**0**.016*	0.206	0.208
LDL (mg/dl)	0.266	0.102	0.188	0.251
Visfatin (ng/ml)			0.236	0.149
FGF-21 (pg/ml)	0.236	0.149		

N.B.: *p* < 0.01 = highly significant differences, *p* < 0.05 = Significant differences. *SBP* systolic blood pressure, *DBP* diastolic blood pressure, *NC* neck circumference, *WC* waist circumference, *HC* hip circumference, *FBG* fasting blood glucose, *HOMA-IR* homeostasis model assessment-insulin resistance, *TG* triglycerides, *HDL* high-density lipoprotein, *LDL* low-density lipoprotein, *FGF-21* fibroblast growth factor 21.

*p < 0.05 = significant differences; ** p < 0.01 = highly significant differences.

## Discussion

In the current study, the obese children had highly significant higher values than control ones regarding all the studied clinical and anthropometric parameters. Regarding laboratory investigations, they also had significant higher values regarding FBG, Insulin, HOMA-IR, Visfatin, FGF-21 and C-Peptide, significant lower value regarding Cholesterol and HDL, and insignificant differences regarding LDL and TG.

Coinciding with current laboratory findings, Li et al.^18^, declared that the obese group had high circulating FGF-21, and it was characterized by elevated fasting insulin and HOMA-IR. Martin et al.^19^, also found that HOMA-IR were significantly higher in obese children and adolescents than normal weight ones. Mohamed et al.^20^, also found that anthropometric variables (weight, BMI, WC and HC) were significantly higher, and HDL level was significantly lower in obese group than normal weight (*P* value < 0.05). The significant lower value of Cholesterol among obese than control children can be explained by the fact that total cholesterol levels in the blood are greatly affected by a person’s food intake. Diets high in saturated fat and carbohydrates can raise the levels of total cholesterol in the blood stream^21^.

In contrary to the current results; which reported insignificant differences between obese and control children regarding LDL and TG; Küme et al.^1^ and Martin et al.^19^, found significantly higher triglycerides and LDL-C in obese children, adolescents and adults. Martin et al.^19^, also reported significant higher total cholesterol, and insignificant differences in insulin and blood glucose in obese and normal weight children and adolescents. This can be attributed to age differences as well as the effects of puberty. Li et al.^18^, found elevated triglycerides levels among the obese group.

The current study reported that obese children had significant higher values of serum Visfatin and FGF21 than control ones. In addition, Visfatin and FGF-21 had highly significant positive correlations with each other among the obese group and insignificant correlation with each other among the control group.

In line to current results, significant higher serum FGF-21 levels in obese children than in lean ones was reported previously by Zhang et al.^12^, Baek et al.^10^, and Christaki et al.^22^. Moreover, Baek et al.^10^, found that serum FGF21 levels were also higher in obese children with metabolic syndrome than children without.

Many authors as Elkabany et al.^23^, and Serbis et al.^24^, have concluded that, in obese children group, the serum Visfatin level was higher than that in control group one. In addition, Catalán et al.^25^, reported that circulating Visfatin concentrations and mRNA expression levels in peripheral blood cells were increased in patients with obesity and are related to inflammation, lipid metabolism and hepatic enzymes suggesting a potential involvement in fatty liver disease and in the obesity-associated inflammatory state.

In the present study; Among obese children; both Visfatin and FGF-21 had significant negative correlations with BMI and HC, and insignificant correlations with FBG, insulin, HOMA-IR, and lipid profile; except that serum FGF-21 had highly significant positive correlation with HDL. While among control group, both serum Visfatin or FGF-21 had significant positive correlation with Cholesterol, and insignificant correlations with all the studied clinical and anthropometric parameters, insulin, HOMA, triglycerides and LDL. In addition, serum Visfatin had significant positive correlation with HDL, and significant negative correlation with FBG.

Concurrent with the current results, Reinehr et al.^11^, found that FGF-21 was not related to any parameter of metabolic syndrome in obese children. In the control group, Christaki et al.^23^, have also reported insignificant associations between the metabolic biomarkers and the levels circulating FGF-21, whereas the levels of serum FGF-21 was significantly correlated with the levels of HDL (r =  − 0.294, *P* < 0.05) in the obese group. In contrary to current results, Zhang et al.^12^, found that serum FGF-21 correlated positively with fasting insulin, and triglycerides but negatively with HDL, after adjusting for age and BMI. Christaki et al.^23^, found that FGF21 levels were negatively correlated with insulin and HOMA-IR levels after adjusting for age, gender, puberty and lifestyle factors in the obese group. In addition, Akduman et al.^26^, found no relation between FGF-21 level and age, body weight, BMI, waist circumference, hip circumference, fasting blood sugar, fasting insulin, total cholesterol, HDL-C, LDL-C, in obese and control groups (*P* > 0.05).

In agreement with our results, Ugur et al.^27^, have concluded that, obese group with metabolic syndrome has shown statistically significant negative correlation between Visfatin and BMI (*P* < 0.05). Kamińska et al.^28^, in addition they did not find any significant association between Visfatin levels and any of the following parameters (Weight, height, BMI, WC, HC, WHR and FBG) in the control group. Elkabany et al.^24^, found significant positive correlations between serum Visfatin and total cholesterol, and insignificant correlations with blood pressure or fasting insulin. Ooi et al.^29^ found that serum Visfatin correlated with some obesity markers: BMI, percentage body fat, and fasting triglyceride level. Moreover, increased Visfatin level was recorded among obese children, who had abnormal glucose tolerance and NAFLD. So, they established the association between Visfatin and its genetic variants with the obesity-related morbidities and adverse cardio metabolic parameters.

In contrary to current results, Alnowihi et al.^30^, found that Visfatin levels had significant positive correlations with waist and hip circumferences, BMI, blood pressure (DBP and SBP), insulin, HOMA, and LDL-C levels, and significant negative correlation with HDL-C. Elkabany et al.^24^, reported that Visfatin had significant positive correlations with BMI and waist circumference, and insignificant correlation with FBG. While Ugur et al.^27^, found significant negative correlation between Visfatin and waist circumference in the obese group with metabolic syndrome.

## Conclusion

Among the obese prepubertal Egyptian children, Fibroblast Growth Factor-21 (FGF21) and Visfatin were highly significant higher than control ones, and they have highly significant positive correlations with each other. Both of them are related to the obesity markers, but they cannot be used as potential predictors for metabolic disturbance in obese prepubertal children; as both of them had insignificant correlations with the metabolic risk factors (WC, BP, FBG, insulin, HOMA-IR, and lipid profile).

## Data Availability

The datasets used and/or analysed during the current study are available from the corresponding author on reasonable request, after taking the permission of our institute “National Research Centre”.
